# Supplementary material for: Vesicular transport mediates the uptake of cytoplasmic proteins into mitochondria in Drosophila melanogaster
Source: Nat Commun. 2020 May 22;11:2592. doi: 10.1038/s41467-020-16335-0 (PMC7244744; doi:10.1038/s41467-020-16335-0)
Supplement: Supplementary file 1 — Supplementary Information [file 41467_2020_16335_MOESM1_ESM.pdf]

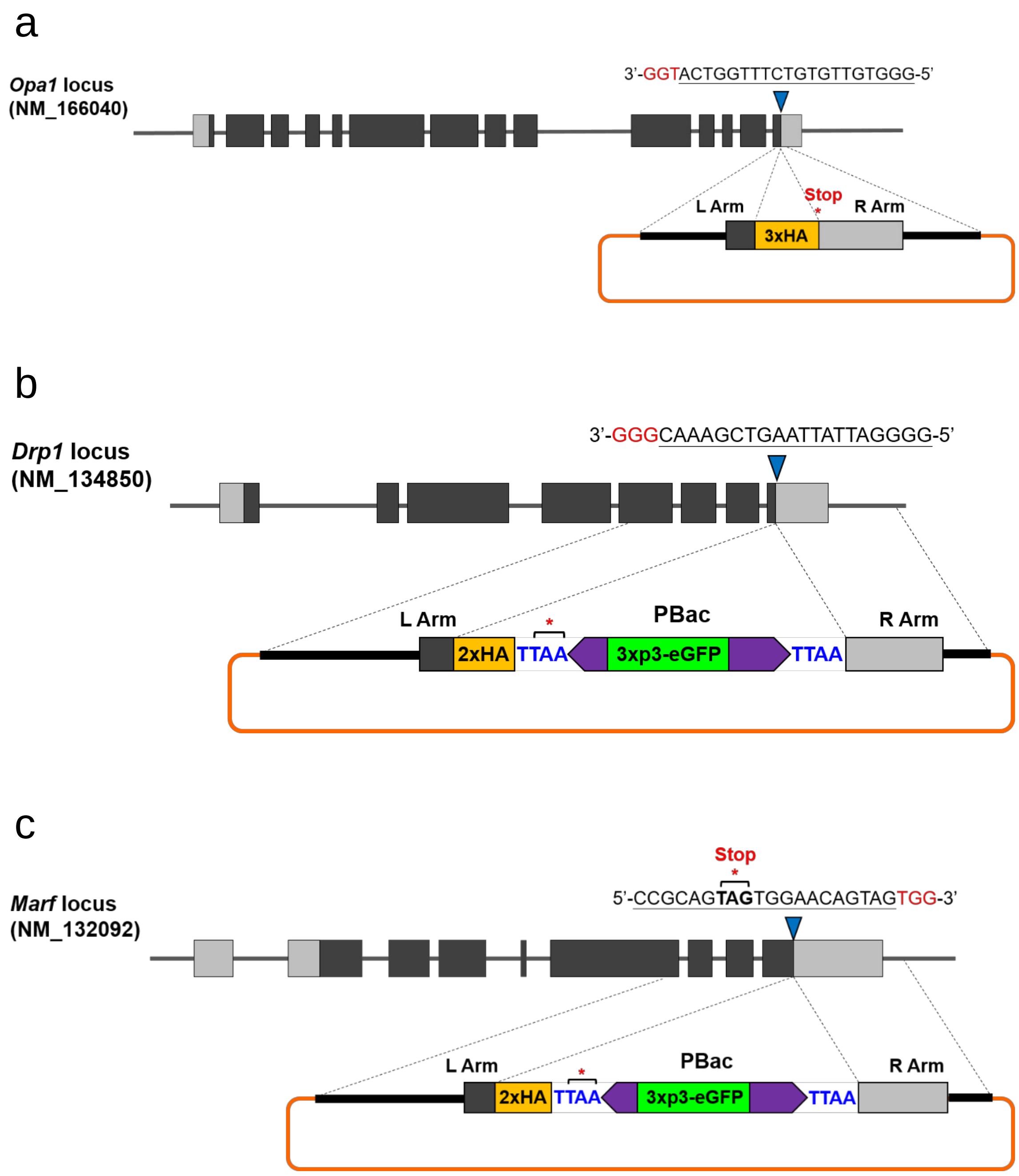

Supplementary Figure 1. CRISPR/Cas9-mediated HA-tagged-in transgenic flies for genes affecting mitochondrial dynamics.

(a–c) HA sequence was knocked-in the C-terminus of *Opa1* **(a)**, *Drp1* **(b)**, and *Marf* **(c)**. HA expression could then be controlled using their respective promoters. eGFP is a selective marker driven by eye-specific promoter 3xp3 for screening.

Supplementary Figure 1

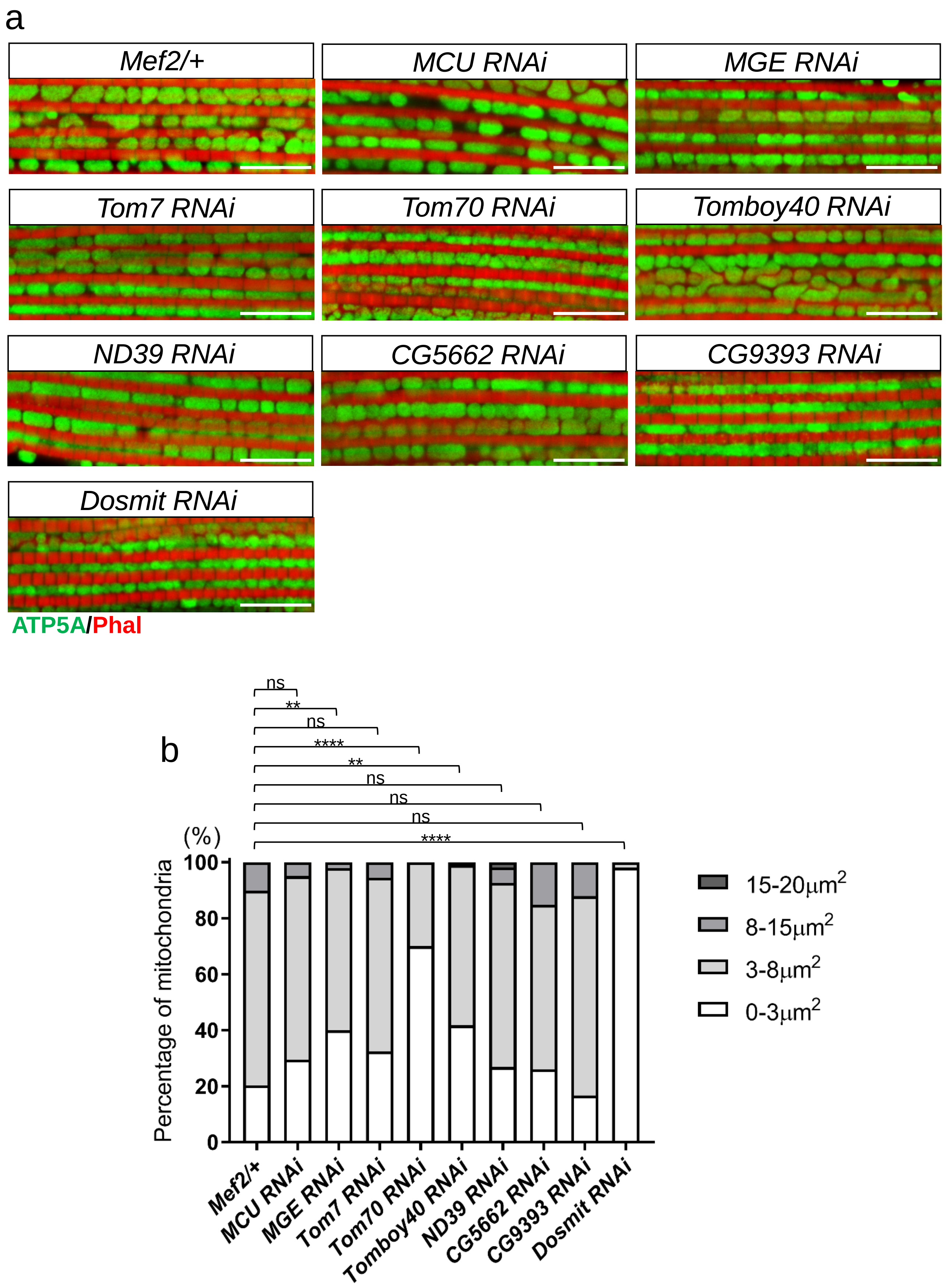

Supplementary Figure 2

Supplementary Figure 2. Screening of mitochondrial morphology by knocking down nine mitochondria-associated proteins.

(a) *Mef2/+*, *MCU RNAi*, *MGE RNAi*, *Tom7 RNAi*, *Tom70 RNAi*, *Tomboy40 RNAi*, *ND39 RNAi*, *CG5662 RNAi*, *CG9393 RNAi*, and *Dosmit RNAi* fly muscle stained with anti-ATP5A (green) and phalloidin (red). Scale bars: 10 µm. **(b)** Distribution of mitochondrial sizes for one-week-old *Mef2/+*, *MCU RNAi, MGE RNAi, Tom7 RNAi, Tom70 RNAi*, *Tomboy40 RNAi, ND39 RNAi, CG5662 RNAi, CG9393 RNAi,* and *Dosmit RNAi* flies. N=118, 61, 95, 108, 97, 103, 108, 92, 66 and 107 from left to right bar. Statistical test: chi-square test (** $p<0.01$; **** $p<0.0001$; ns: $p=0.1412$, 0.0756, 0.317, 0.401, 0.7161 from left to right comparison). Source data are provided as a Source Data file.

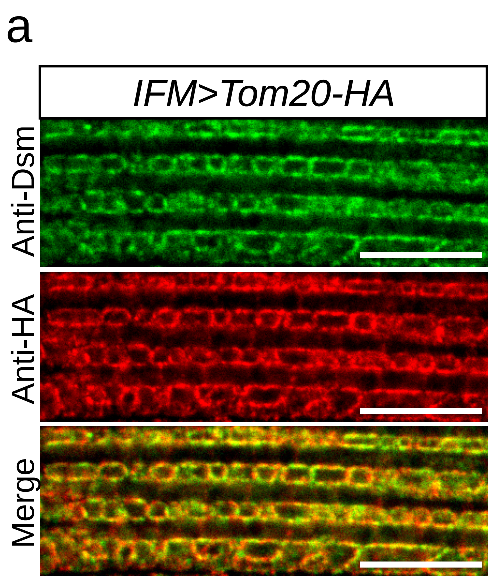

Supplementary Figure 3. Dosmit was localized on the mitochondrial outer membrane.

(a) Tom20-HA was ectopically expressed in muscle as a mitochondrial outer marker. Dosmit (Dsm, green) was colocalized with Tom20-HA (red). Scale bar: 10 µm.

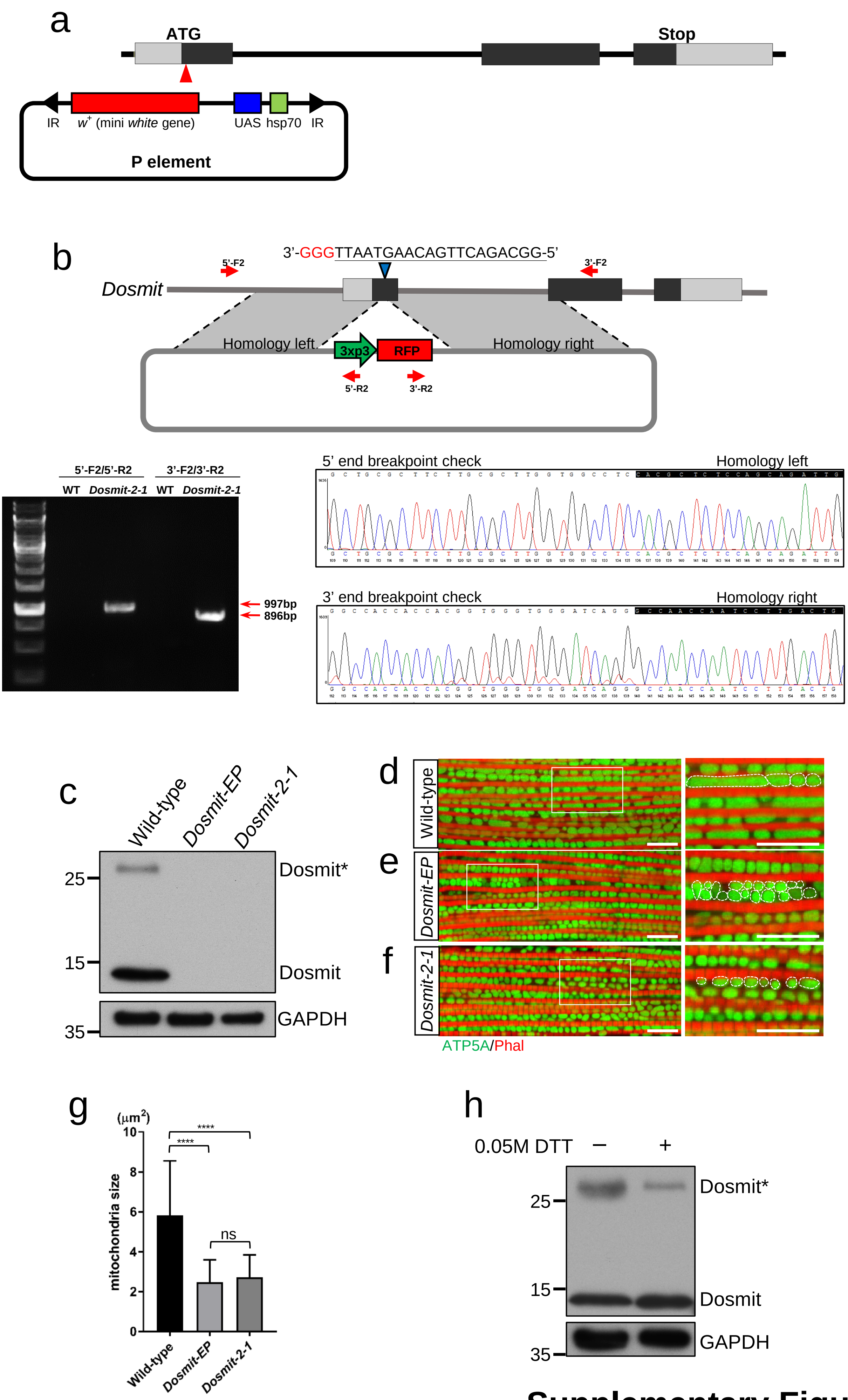

Supplementary Figure 4

Supplementary Figure 4. CRISPR/Cas9-mediated Dosmit-null mutant showed fragmented mitochondria like *Dosmit-EP*, and Dosmit* is a dimer form of Dosmit.

(a) P-element integrated into the first exon of *Dosmit-EP*. **(b)** Design of CRISPR/Cas9-generated *Dosmit-2-1* mutant, and confirmation of Dosmit disruption via PCR. **(c)** Western blot showing expression levels of Dosmit for wild-type, *Dosmit-EP*, and *Dosmit-2-1* flies. Dosmit*: dimer form of Dosmit. GAPDH: loading control. Mitochondria from **(d)** wild-type, **(e)** *Dosmit-EP*, and **(f)** *Dosmit-2-1* flies. The green channel indicates ATP5A staining, and the red channel indicates phalloidin staining of F-actin in muscle. Scale bars: 10 μm. **(g)** Mitochondrial size (mean ± SD) in wild-type, *Dosmit-EP*, and *Dosmit-2-1* flies. N=112, 89 and 96 from left to right bars. Statistical test: Two-tailed Mann–Whitney *U* test (****$p<0.0001$; ns, not significant, $p=0.0936$). **(h)** Western blot of wild-type flies showing expression levels of Dosmit in both monomeric and dimeric (Dosmit*) forms under reducing (+) and non-reducing (–) conditions. Source data are provided as a Source Data file.

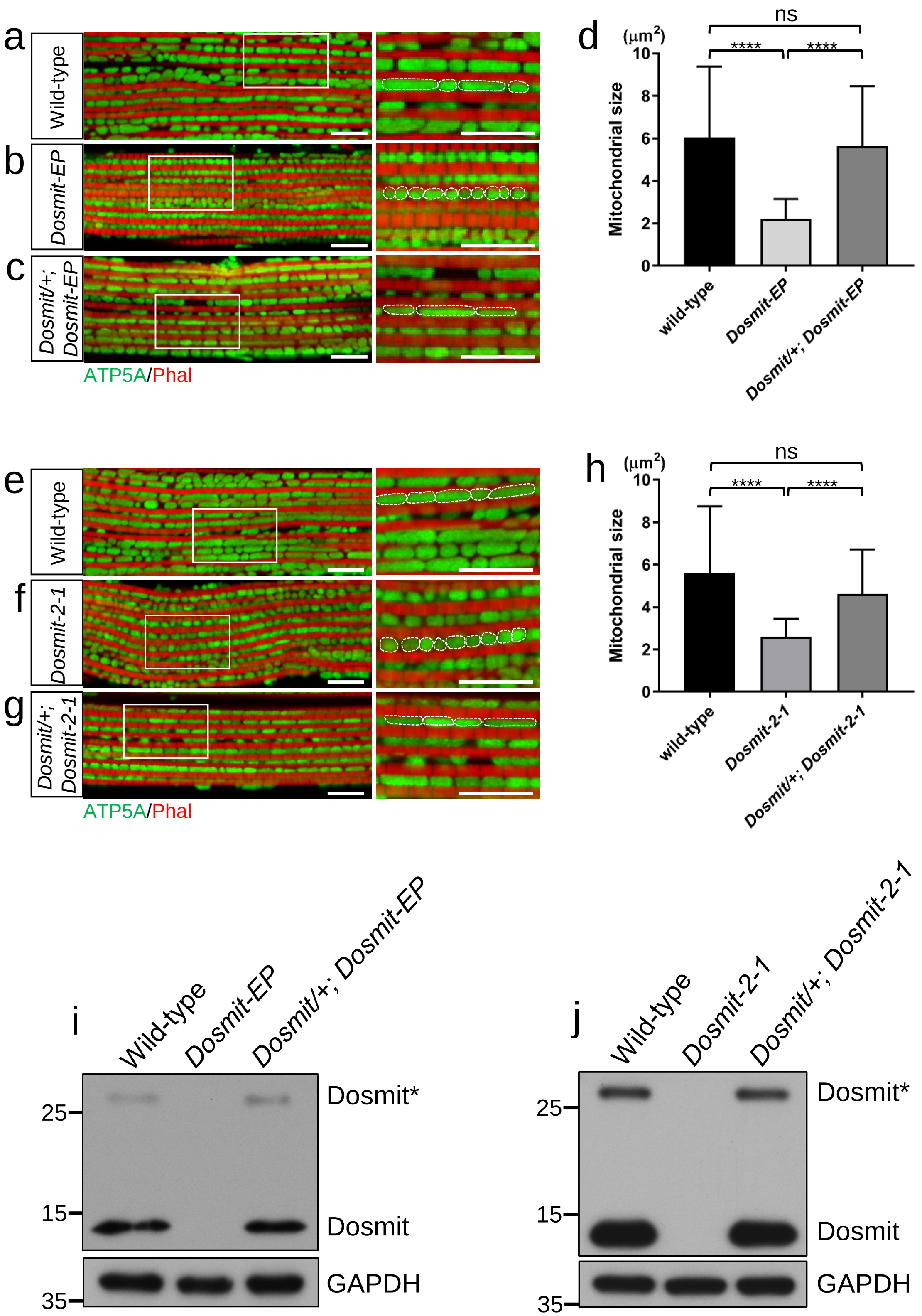

Supplementary Figure 5

Supplementary Figure 5. The genomic sequence of Dosmit could suppress the circular mitochondrial phenotype of *Dosmit-EP* or *Dosmit-2-1* flies.

(a–g) Mitochondria from wild-type **(a, e)**, *Dosmit-EP* **(b)**, *Dosmit/+; Dosmit-EP* **(c)**, *Dosmit-2-1* **(f)**, and *Dosmit/+; Dosmit-2-1* **(g)** flies. The green channel indicates ATP5A staining and the red channel indicates phalloidin staining of F-actin in muscle. Scale bars: 10 µm. **(d)** Quantification of mitochondrial sizes for wild-type, *Dosmit-EP*, and *Dosmit/+; Dosmit-EP* flies (mean ± SD). N=283, 158 and 181 from left to right bars. Statistical test: Two-tailed Mann–Whitney *U* test (****p<0.0001; ns: p=0.3128). **(h)** Quantification of mitochondrial sizes for wild-type, *Dosmit-2-1, and Dosmit/*+; *Dosmit-2-1* flies (mean ± SD). N=70, 63 and 76 from left to right bars. Statistical test: Two-tailed Mann–Whitney *U* test (****p<0.0001; ns: p=0.0549). **(i, j)** Western blot showing Dosmit re-expression in genetically rescued flies. Source data are provided as a Source Data file.

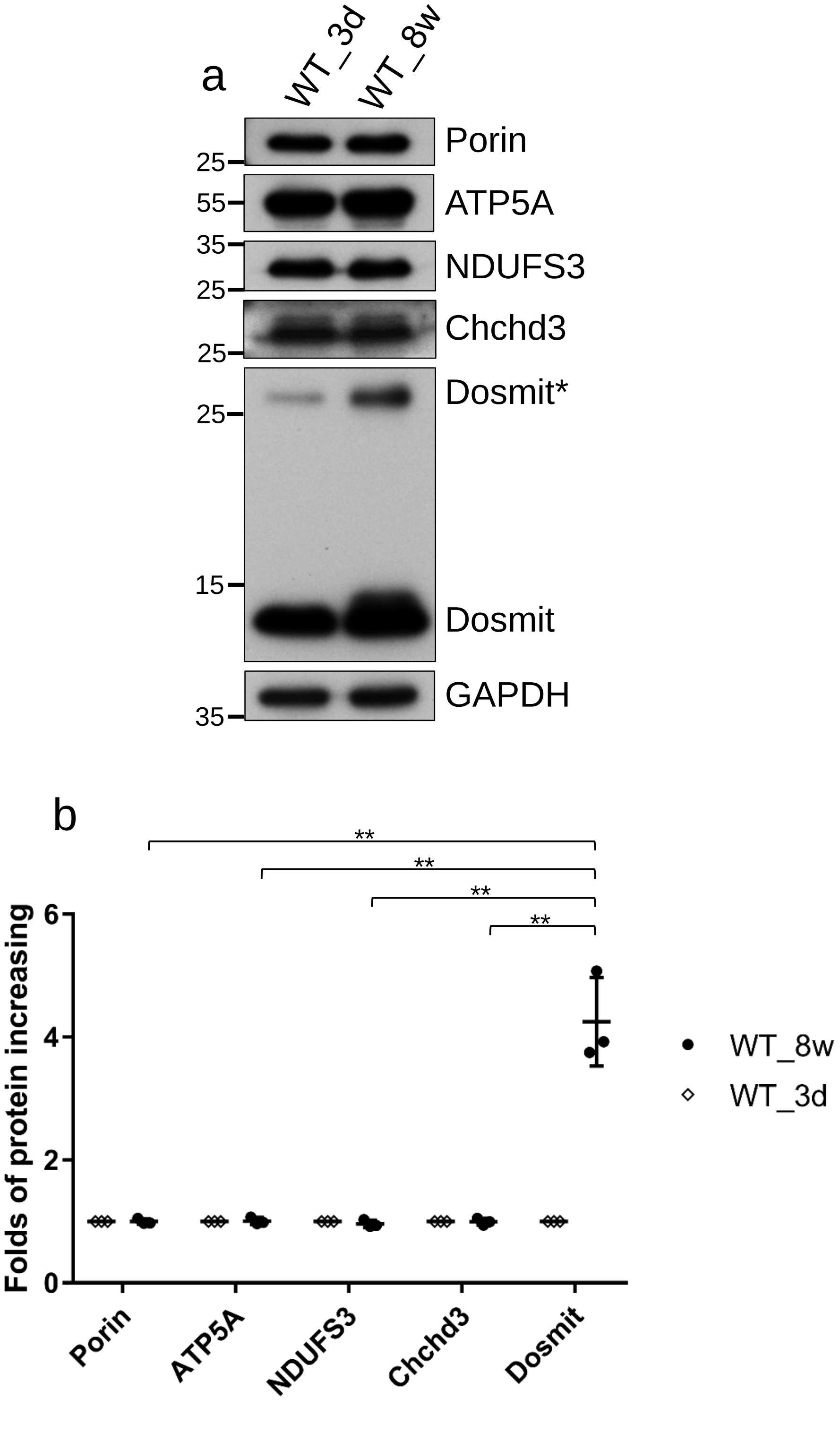

Supplementary Figure 6. Dosmit level increased during aging but not other mitochondrial proteins.

(a) Western blotting of one- and eight-week-old wild-type flies. **(b)** Fold-change of Porin, ATP5A, NDUFS3, Chchd3, and Dosmit in three-day- and eight-week-old w^{1118} flies (mean ± SD). N=3, Statistical differences between ages are starred (Two-tailed Mann-Whitney *U* test: ** p<0.01). Source data are provided as a Source Data file.

Supplementary Figure 6

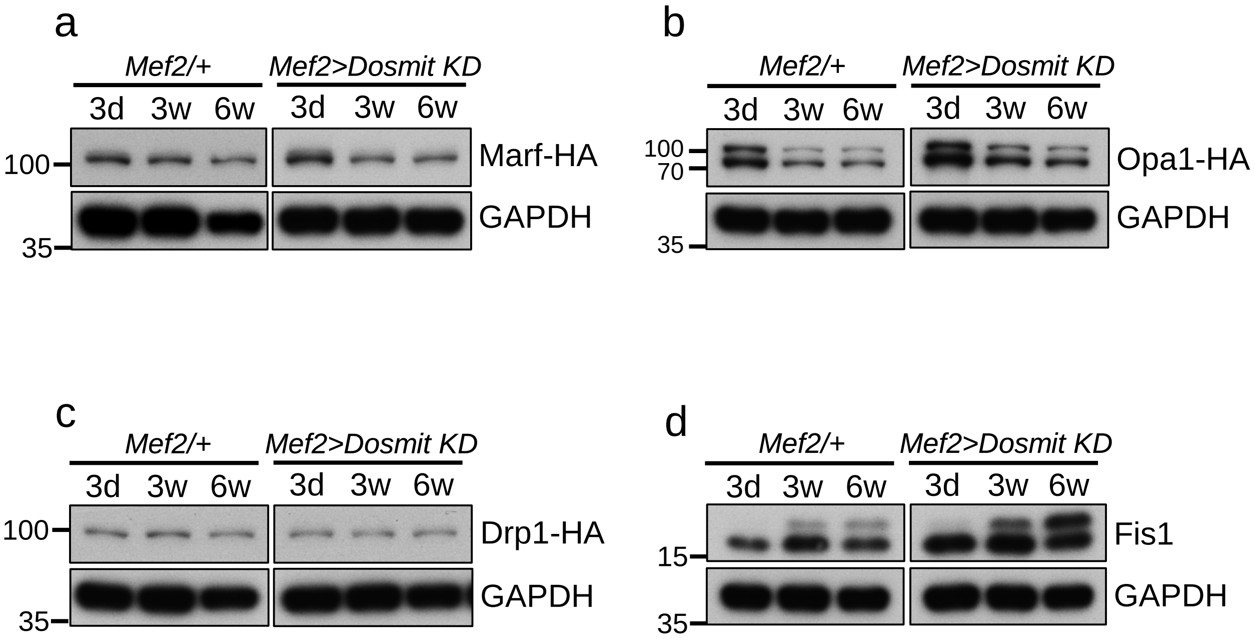

Supplementary Figure 7. The protein level of mitochondrial dynamics proteins changed during aging in independent of Dosmit.

(a–d) The patterns of Marf **(a)**, Opa1 **(b)**, Drp1 **(c)**, and Fis1 **(d)** protein levels in Dosmit-knockdown flies during aging were not significantly different from those in *Mef2/+* flies. Source data are provided as a Source Data file.

Supplementary Figure 7

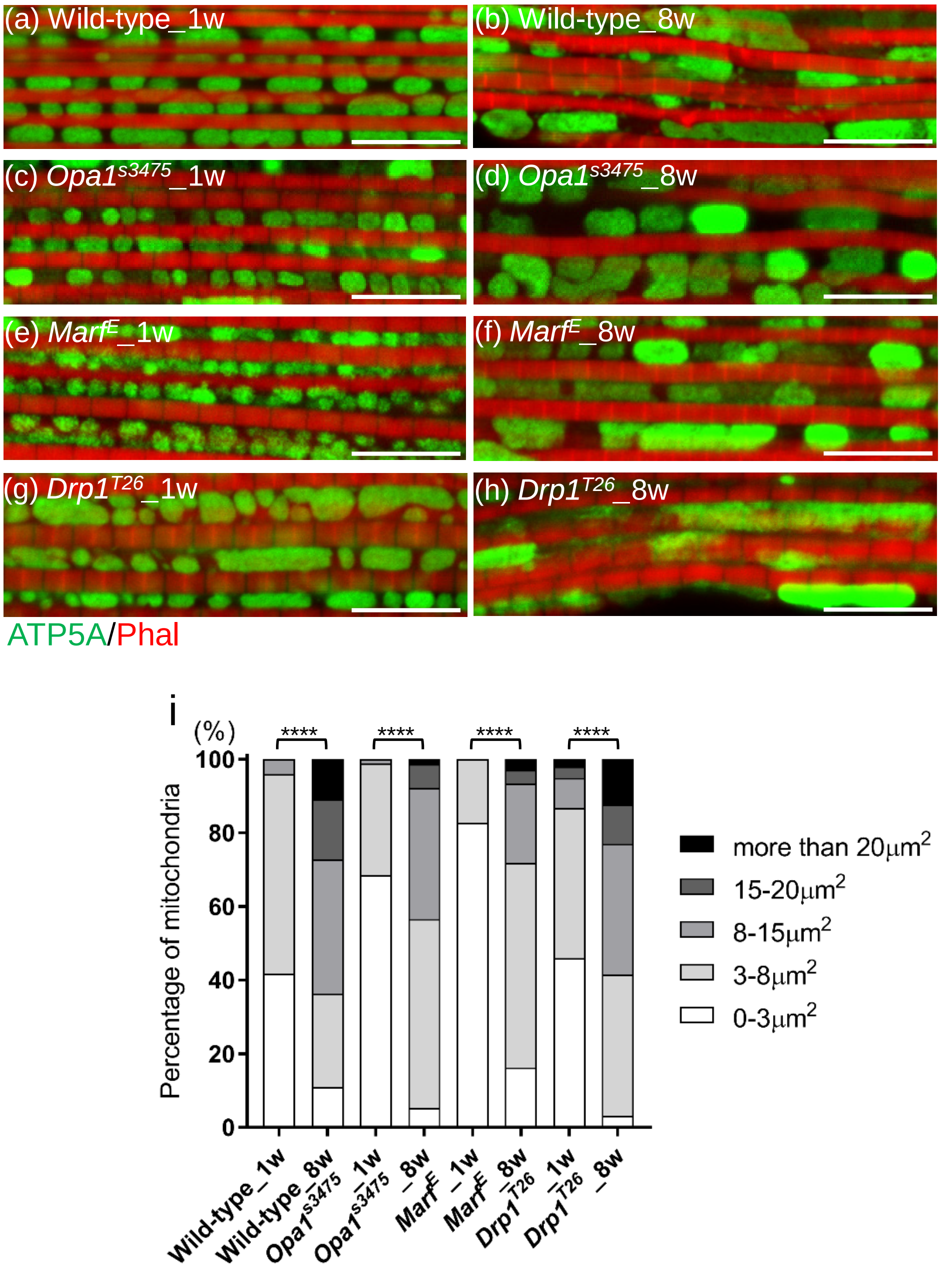

Supplementary Figure 8. The size of mitochondria still enlarged in aged *Drp1*^{T26}, *Opa1*^{s3475} and *Marf*^{E} fly lines.

(a, b) Wild-type flies exhibited mitochondrial enlargement with aging. **(c–f)** One-week-old *Opa1*^{s3475} and *Marf*^{E} flies exhibited more fragmented mitochondria than wild-type flies did, but their mitochondrial size had increased by the eight-week-old stage. **(g, h)** One-week-old *Drp1*^{T26} exhibited more fused mitochondria than wild-type flies did, and their mitochondrial size had increased by the age of eight weeks old**.** Scale bars: 10 μm. **i)** Categorization of mitochondrial size for one- and eight-week-old wild-type, *Opa1*^{S3475},*Marf*^{E}, and *Drp1*^{T26} flies. N=122, 55, 89, 76, 122, 135, 98 and 65 from left to right bars. Statistical test: chi-square test (****p<0.0001). Source data are provided as a Source Data file.

Supplementary Figure 8

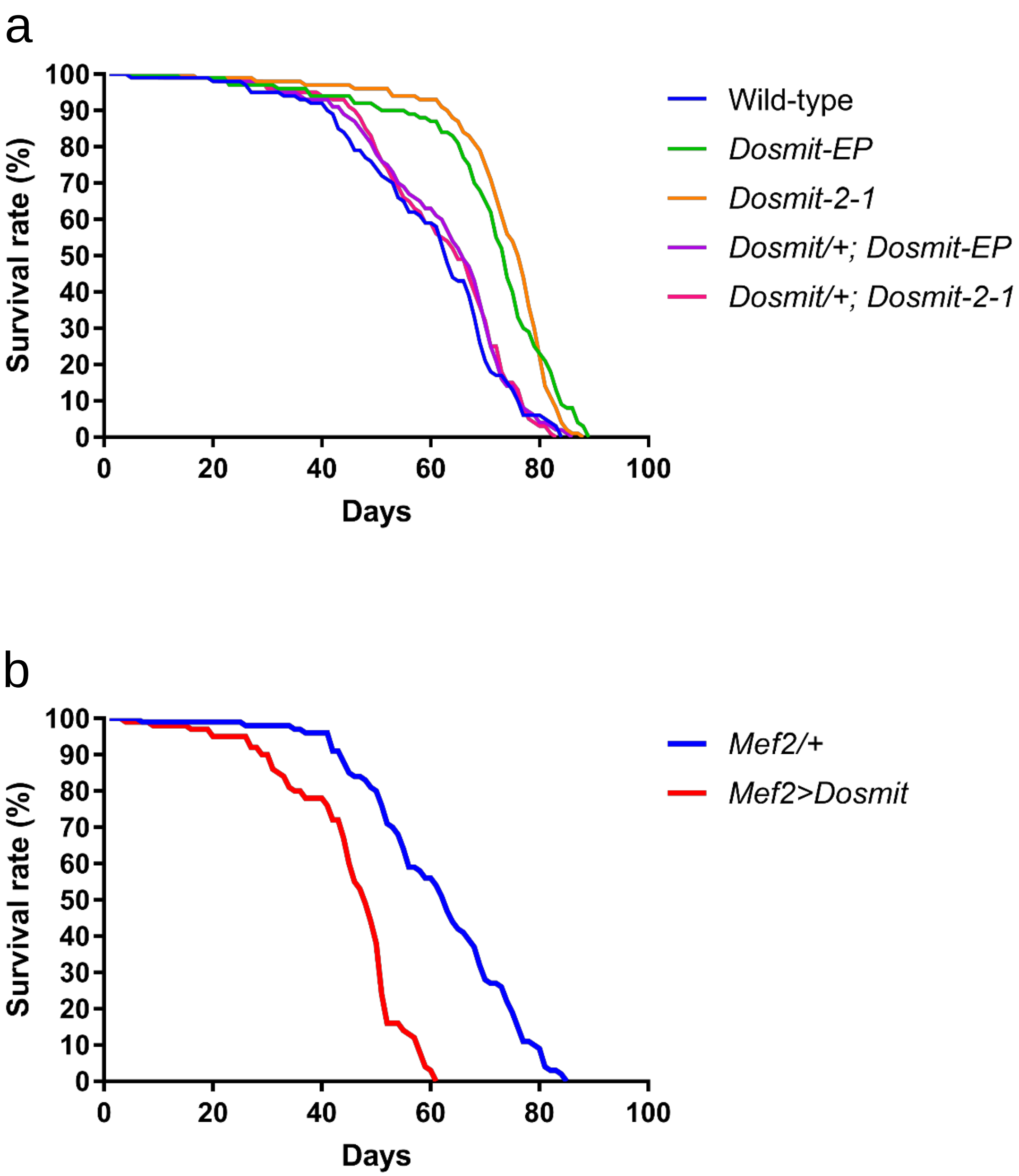

Supplementary Figure 9. Effects of Dosmit overexpression and knock-out on the lifespan of *Drosophila melanogaster*.

(a) Dosmit expression improved the median lifespan of *Dosmit-EP* and *Dosmit-2-1* flies (N = 100). **(b)** Flies that ectopically expressed Dosmit in their muscles exhibited a shorter median lifespan than *Mef2/+* flies (N = 100). The median lifespans were as follows: wild-type, 62.6 d; *Dosmit-EP*, 73.3 d; *Dosmit-2-1*, 76 d; *Dosmit/+; Dosmit-EP*, 65.7 d; *Dosmit/+; Dosmit-2-1*, 64.7 d; *Mef2/+*, 62.3 d; and *Mef2>Dosmit*, 47.8 d. Source data are provided as a Source Data file.

Supplementary Figure 9

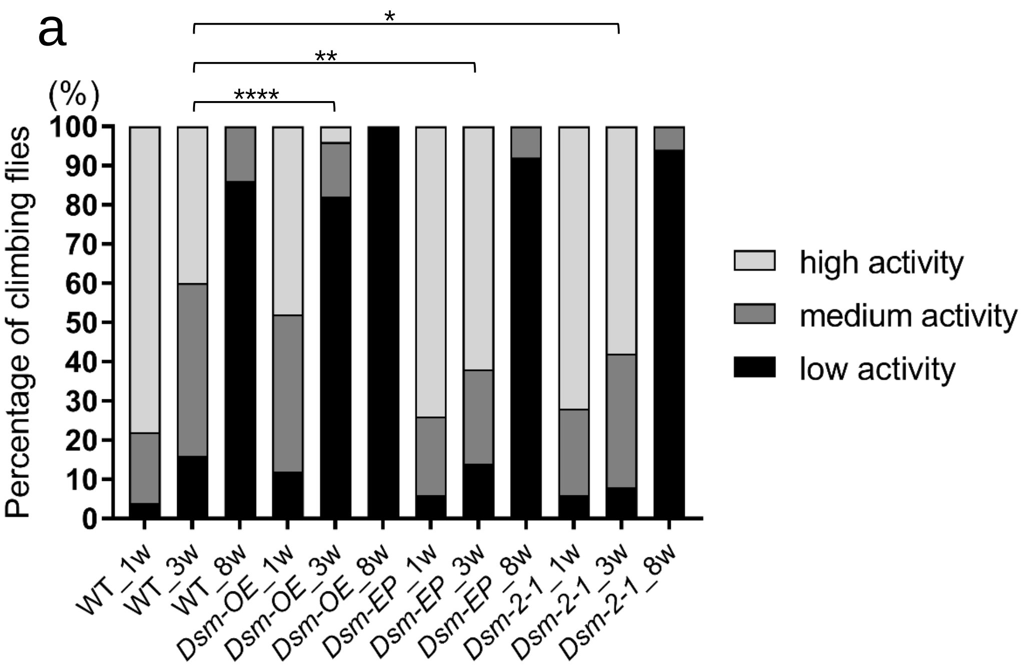

Supplementary Figure 10. In middle-age, Dosmit-mutant flies had a significantly greater climbing ability than wild-type flies.

(a) Percentage of wild-type, Dosmit-overexpressing (*Dsm-OE*), *Dosmit-EP* (*Dsm-EP*), and *Dosmit-2-1* (*Dsm-2-1*) flies categorized as exhibiting low, medium, and high activity. *Dosmit-EP* and *Dosmit-2-1* flies showed significantly more climbing activity than wild-type flies in middle age (three weeks old). N = 100 flies. Statistical test: chi-square test (*p<0.05; **p<0.01; ****p<0.0001). Source data are provided as a Source Data file.

Supplementary Figure 10

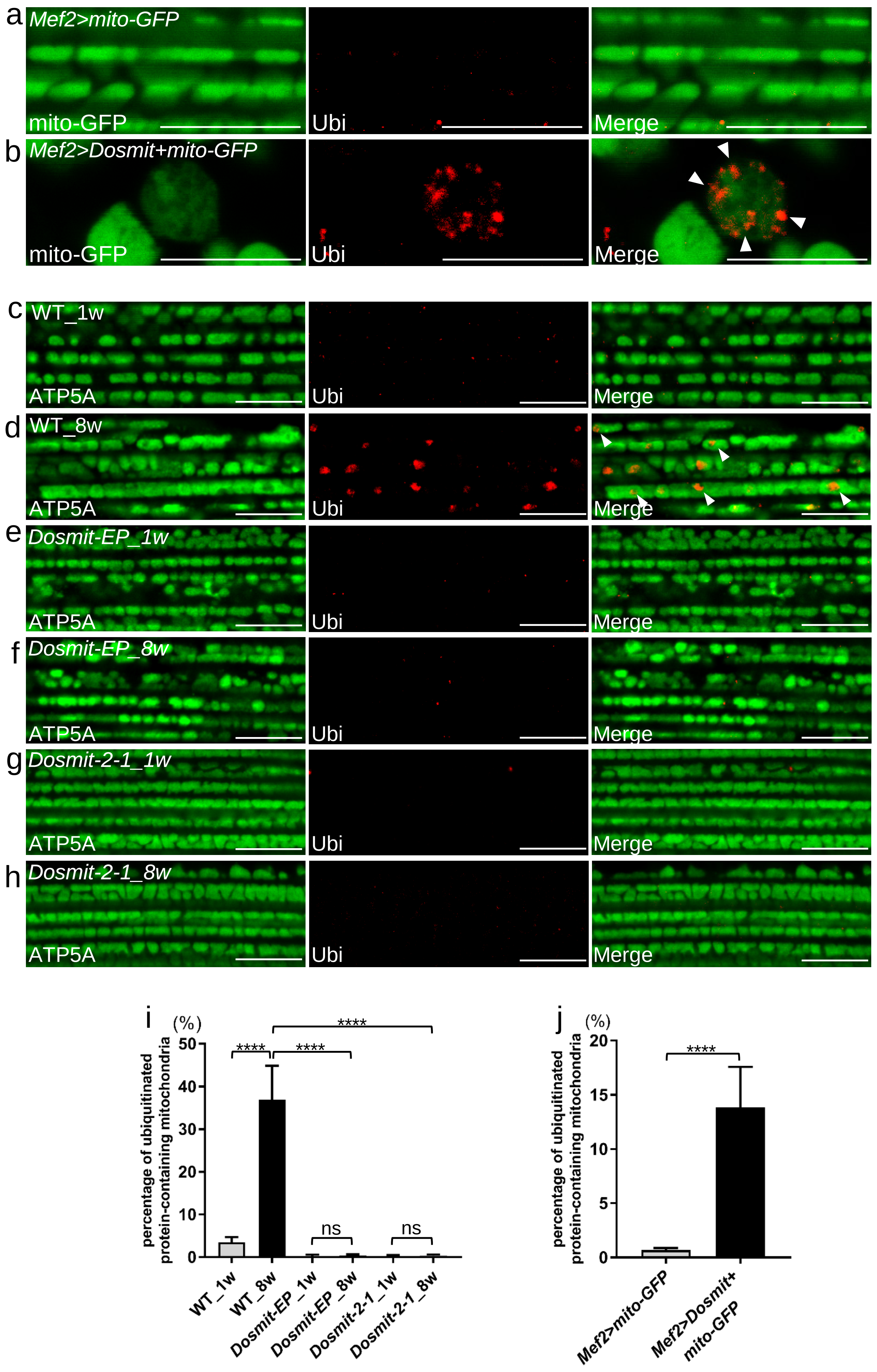

Supplementary Figure 11

Supplementary Figure 11. Ubiquitinated proteins accumulate within the mitochondria in ectopically expressed Dosmit flies and aged wild-type flies.

(a, b) *Mef2>mito-GFP* **(a)** and *Mef2>Dosmit+mito-GFP* **(b)** fly muscle stained with ATP5A (green) and ubiquitin antibody (red). Ubiquitinated proteins accumulated in the mitochondria of *Mef2>Dosmit+mito-GFP* flies. **(c, d)** Ubiquitinated proteins also accumulated in large puncta and were distributed throughout aged wild-type muscle, particularly in the intramitochondrial vesicles (arrowheads). **(e–h)** Ubiquitinated proteins did not form or accumulate in either young or aged *Dosmit-EP* or *Dosmit-2-1* flies. Scale bars: 10 µm. **(i, j)** Quantification of mitochondria in which ubiquitinated protein accumulated (mean ± SD). N=696, 698, 823, 829, 1246 and 859 from left to right bars of (i); N=719 and 189 from left to right bars of (j). Statistical test: Two-tailed Mann–Whitney *U* test (****p<0.0001). Source data are provided as a Source Data file.

Supplementary Figure 11

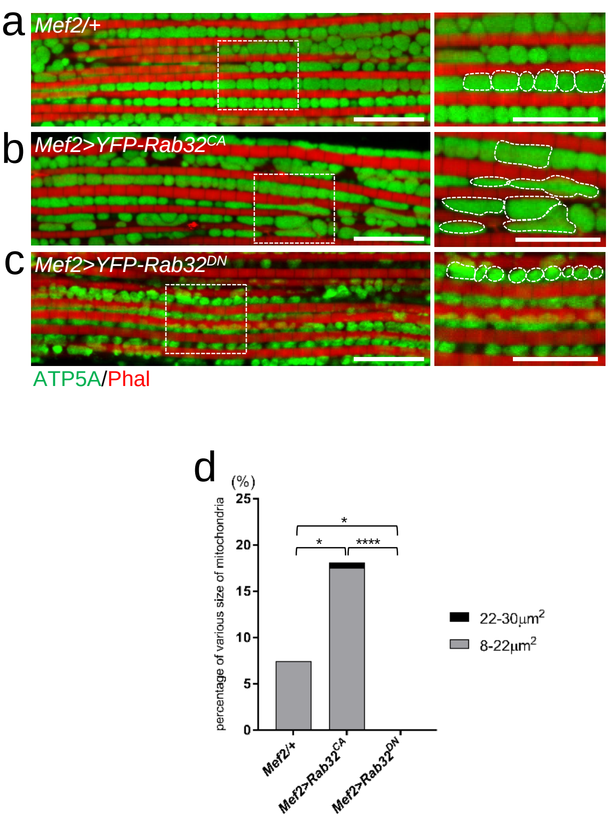

Supplementary Figure 12. Constitutive active or dominant negative forms of Rab32 can up- or downregulate mitochondrial size.

(a) Mitochondria from *Mef2/+* control flies. **(b, c)** Ectopic expression of YFP-Rab32^{CA} **(b)** or Rab32^{DN} **(c)** increased or decreased mitochondrial size, respectively. The green channel indicates ATP5A staining and the red channel indicates phalloidin staining of F-actin in muscle. Scale bars: 10 µm. **(d)** Percentage of mitochondria with a size of 8–22 µm^2 or 22–30 µm^2. N=174, 171 and 197 from left to right bars. Statistical test: chi-square test (*p<0.05; ****p<0.0001). Source data are provided as a Source Data file.

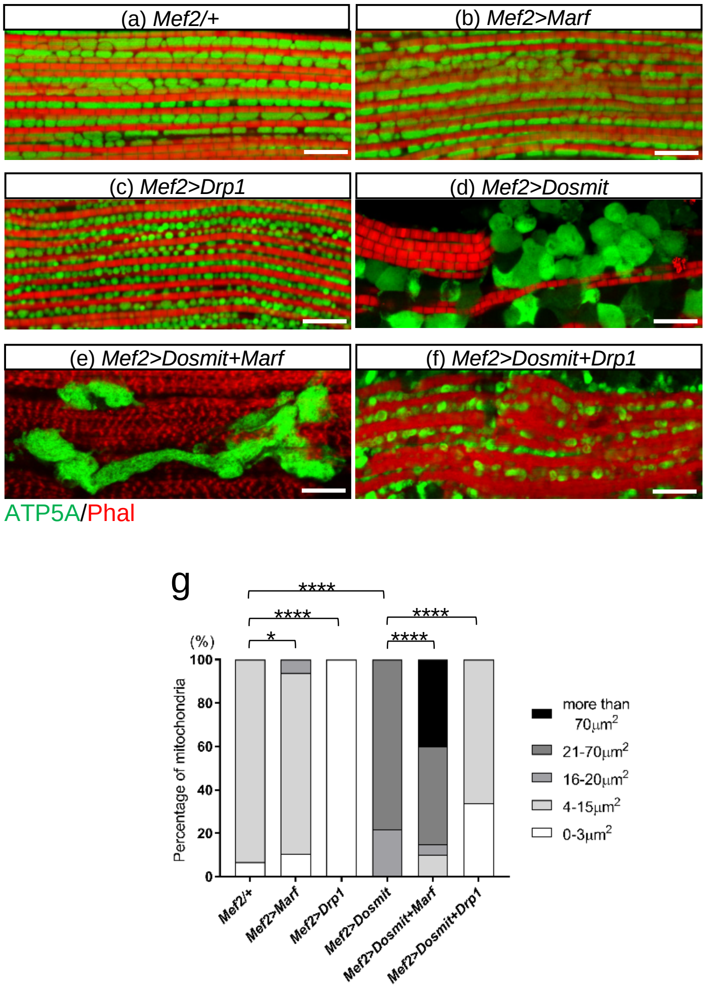

Supplementary Figure 13. Dosmit-induced mitochondrial enlargement could be regulated by Drp1 and Marf.

(a–f) Mitochondria in muscle of Mef2-GAL4 control flies (*Mef2/+*) **(a)**, flies ectopically expressing Marf (*Mef2>Marf*) **(b)**, flies ectopically expressing Drp1 only (*Mef2>Drp1*) **(c)**, flies ectopically expressing Dosmit only (*Mef2>Dosmit*) **(d)**, flies ectopically coexpressing Dosmit and Marf (*Mef2>Dosmit+Marf*) **(e)**, and flies ectopically expressing both Drp1 and Dosmit (*Mef2>Dosmit+Drp1*) **(f)**, showing that Drp1 suppressed Dosmit-induced mitochondrial enlargement. The green channel indicates ATP5A staining and the red channel indicates phalloidin staining of F-actin in muscle. Scale bars: 10 μm. **(g)** Distribution of mitochondrial size classes in the various genetic backgrounds. N= 60, 66, 95, 23, 20 and 62 from left to right bars. Statistical test: chi-square test (*$p<0.05$; ****$p<0.0001$). Source data are provided as a Source Data file.

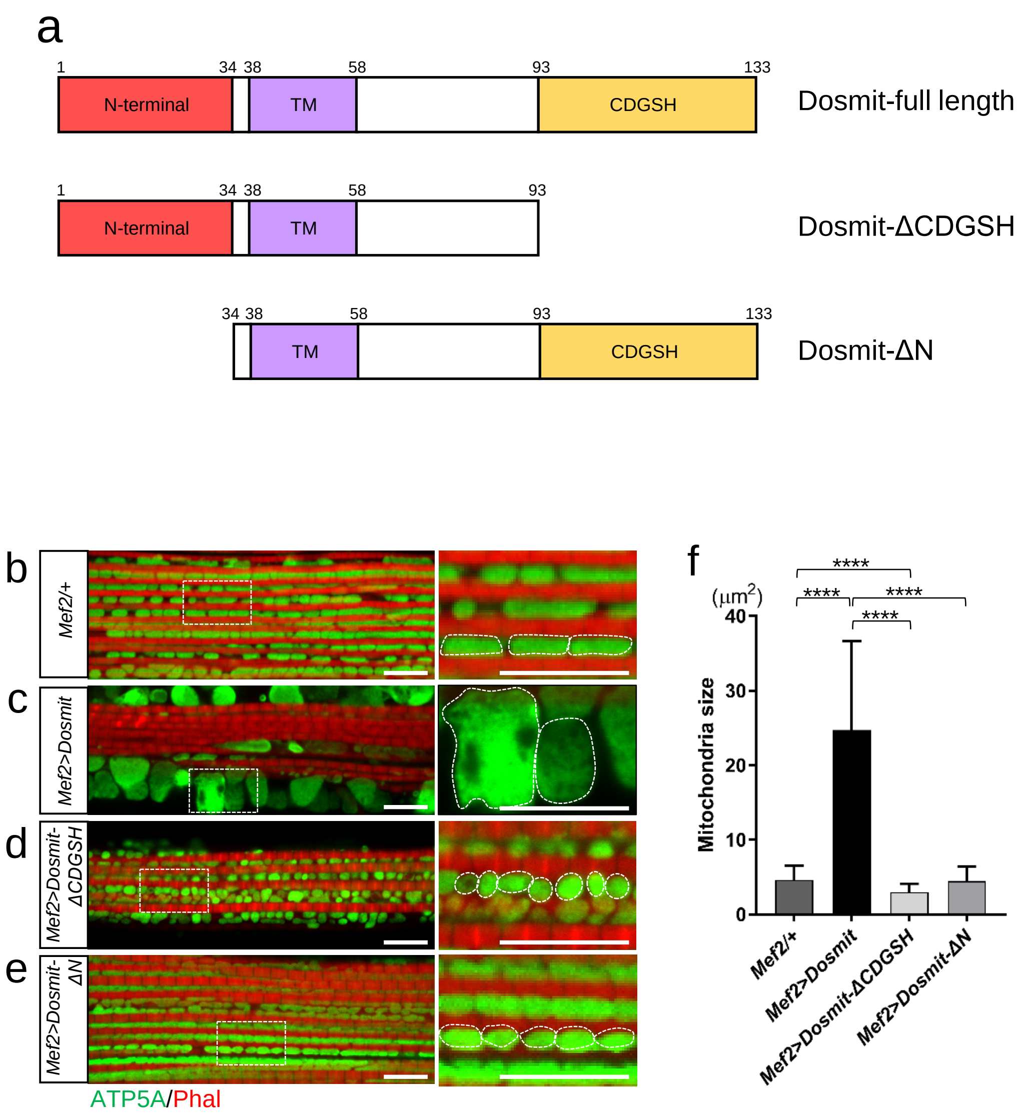

Supplementary Figure 14. The N-terminus and CDGSH domain of Dosmit are required for mitochondrial enlargement.

(a) Illustration of full-length Dosmit protein, as well as the ΔN-terminus and ΔCDGSH forms. **(b–e)** Mitochondria of *Mef2-Gal4* control **(b)**, ectopic expression of Dosmit **(c)**, Dosmit lacking CDGSH **(d)**, and Dosmit lacking N-terminus (**e**). The green channel indicates ATP5A staining and the red channel indicates phalloidin staining of F-actin in muscle. Scale bars: 10 μm. **(f)** Mitochondrial size in *Mef2/+*, *Mef2>Dosmit*, *Dosmit-ΔCDGSH*, and *Mef2>Dosmit-ΔN* flies (mean ± SD). N=136, 35, 96 and 111 from left to right bars. Statistical test: Two-tailed Mann–Whitney *U* test (****$p<0.0001$). Source data are provided as a Source Data file.

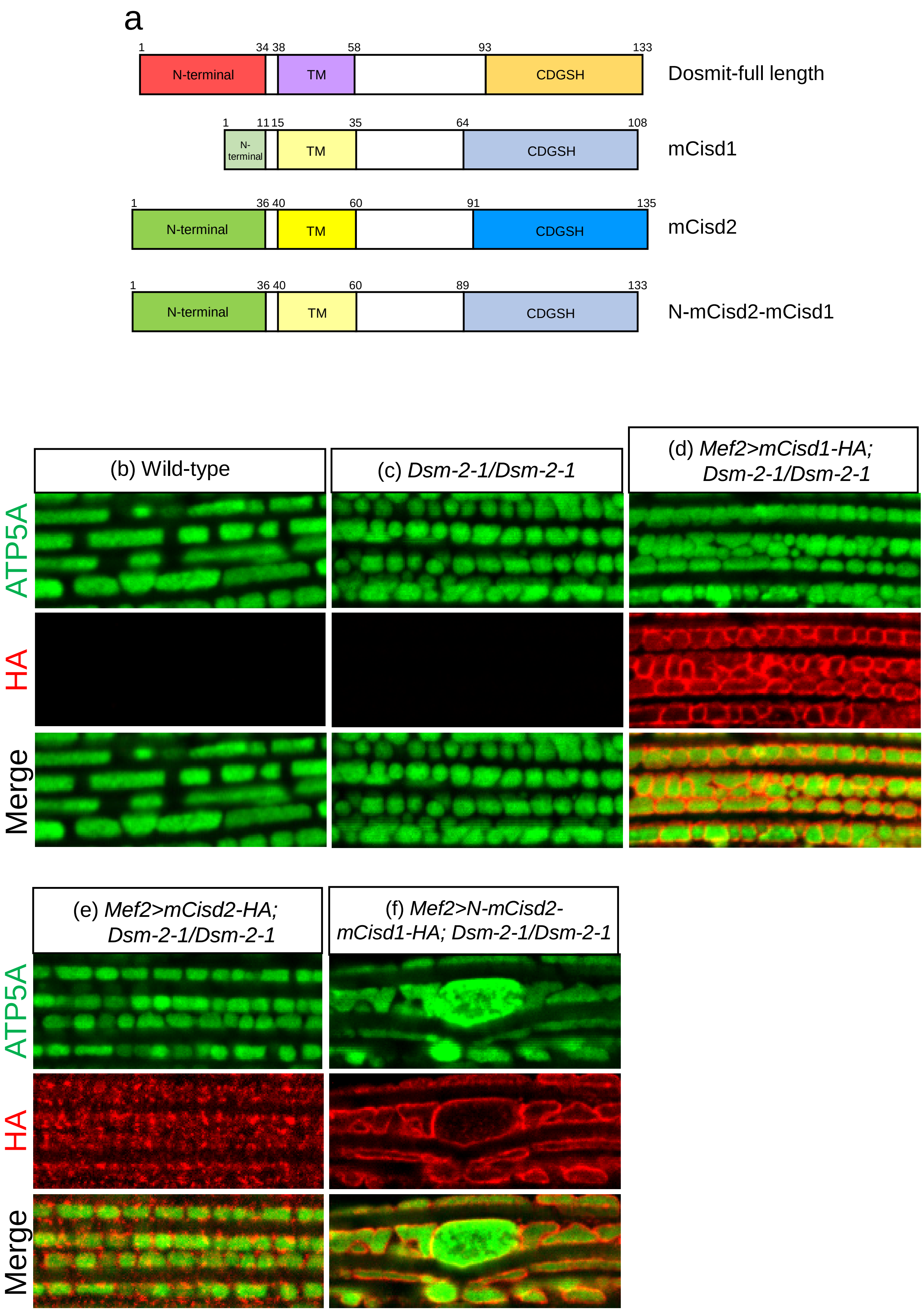

Supplementary Figure 15

Supplementary Figure 15. A hybrid transgene in which the N-terminus of mouse Cisd2 was combined with mouse Cisd1 suppressed fragmented mitochondrial phenotypes in Dosmit-mutated flies.

(a) Construct illustration of the Dosmit (Dsm), mouse Cisd1 (mCisd1), mouse Cisd2 (mCisd2), N-mouse Cisd2-mouse Cisd1 (N-mCisd2-mCisd1) genes. **(b, c)** *Dosmit-2-1* flies have smaller mitochondria than wild-type flies. **(d–f)** Ectopic expression of mCisd1-HA **(d)** and mCisd2-HA **(e)** did not rescue the *Dosmit-2-1* phenotype, but N-mCisd2-mCisd1-HA **(f)** did partially induce mitochondrial enlargement. Scale bars: 10 μm.

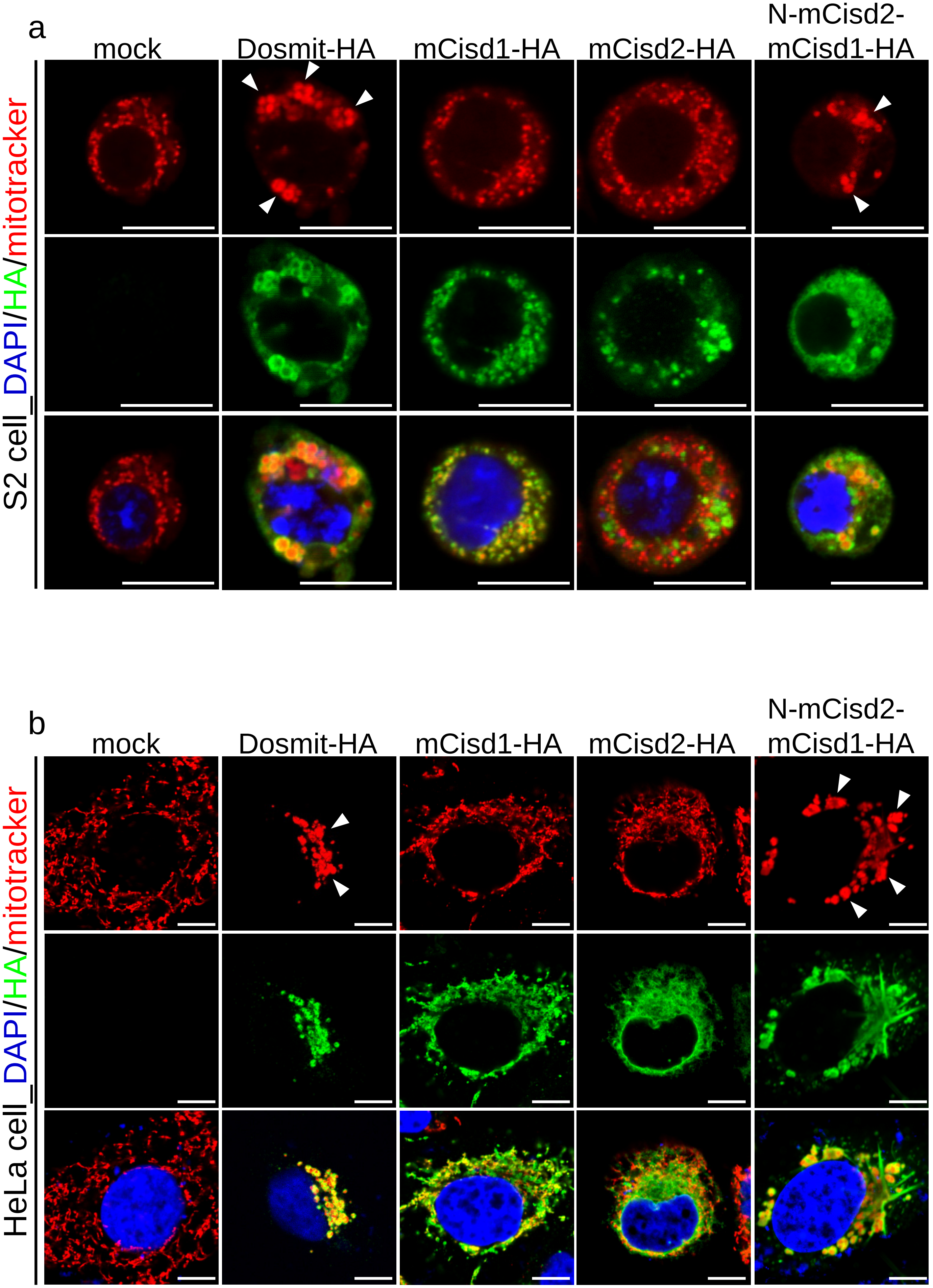

Supplementary Figure 16

c

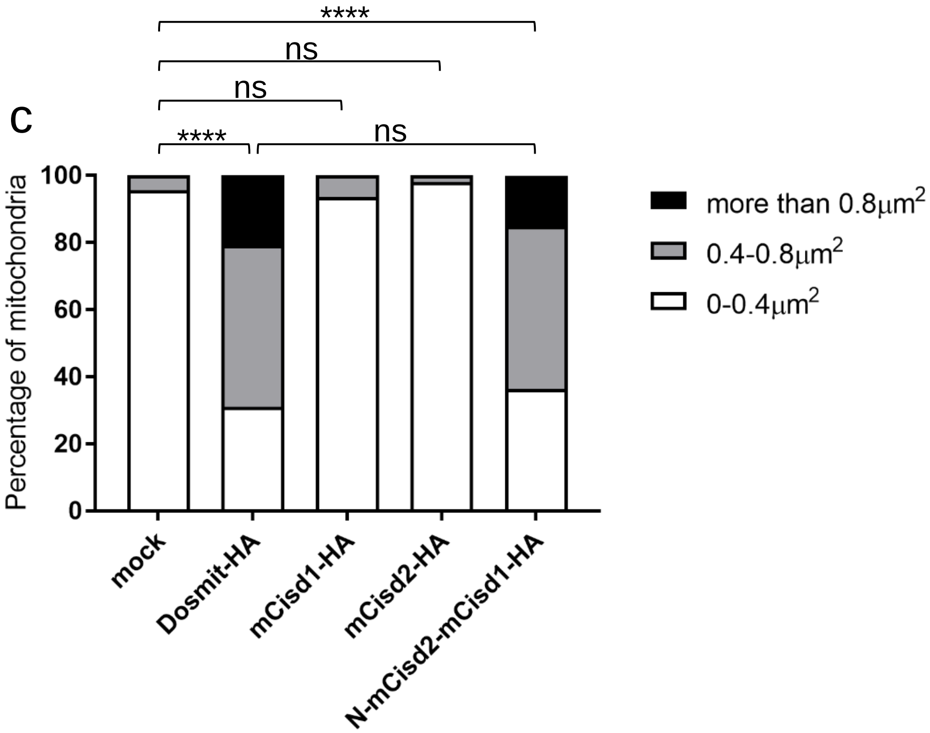

d

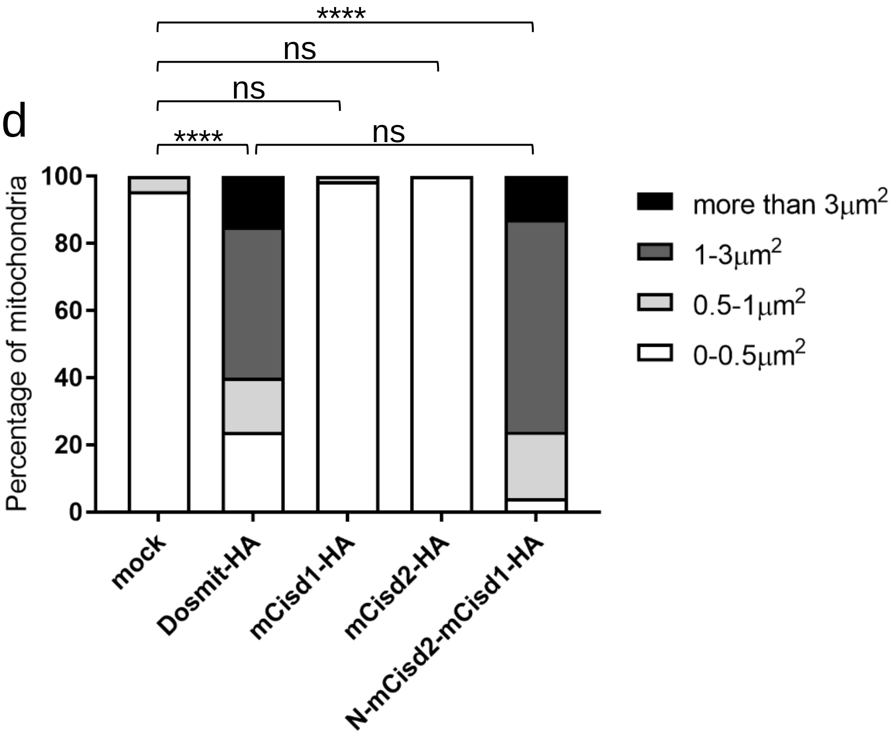

Supplementary Figure 16

Supplementary Figure 16. A hybrid protein in which the N-terminus of mouse Cisd2 was combined with mouse Cisd1 was sufficient for mitochondrial enlargement, but not mCisd1-HA, mCisd2-HA.

(a, b) Dosmit-HA, mCisd1-HA, mCisd2-HA, and N-mCisd2-mCisd1-HA ectopically and respectively expressed in S2 **(a)** and HeLa **(b)** cells stained with MitoTracker (red) and HA (green). Scale bars: 10 μm. **(c), (d)** Quantification of mitochondrial size in S2 **(c)** and HeLa **(d)** cells transfected with Dosmit-HA, mCisd1-HA, mCisd2-HA, or N-mCisd2-mCisd1-HA. N=45, 29, 63, 105 and 33 from left to right bars of (c). (****$p<0.0001$; ns: $p=0.7475$, 0.6827 and 0.5492); N=67, 80, 70, 58 and 71 from left to right bars of (d). (****$p<0.0001$; ns: $p=0.3687$, 0.1212 and 0.8389). Statistical test: chi-square test. Scale bars: 10 μm. Source data are provided as a Source Data file.

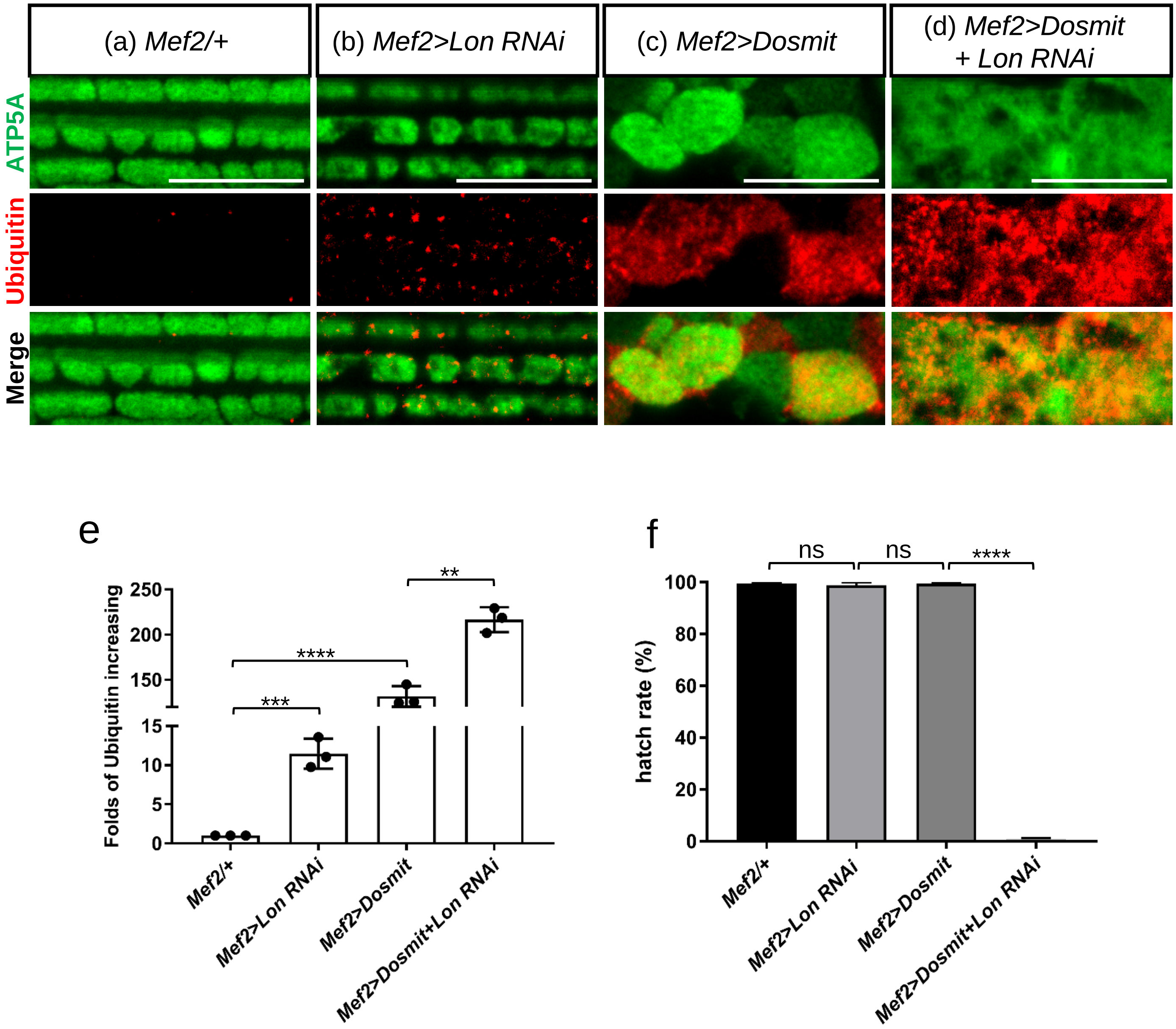

Supplementary Figure 17. Ubiquitinated proteins accumulate in mitochondria when Lon protease is knocked down.
(a, b) Knocking down Lon protease leads to a widespread increase in ubiquitinated proteins. **(c)** Ectopic Dosmit expression causes ubiquitinated protein accumulation in mitochondria. **(d)** Knocking down Lon protease with Dosmit overexpression caused a greater accumulation of ubiquitinated protein than Dosmit overexpression alone. Scale bar: 5 μm. Quantification of **(e)** ubiquitin accumulation (mean ± SD) (Statistical test: Two-tailed student's t test. N = 3. **p<0.01; ***p<0.001 ****p<0.0001) and **(f)** hatch rate (mean ± SD) (N = 100. ****p<0.0001; ns: p=0.3725 and 0.3996 from left to right). Statistical test: Two-tailed student's t test. Source data are provided as a Source Data file.

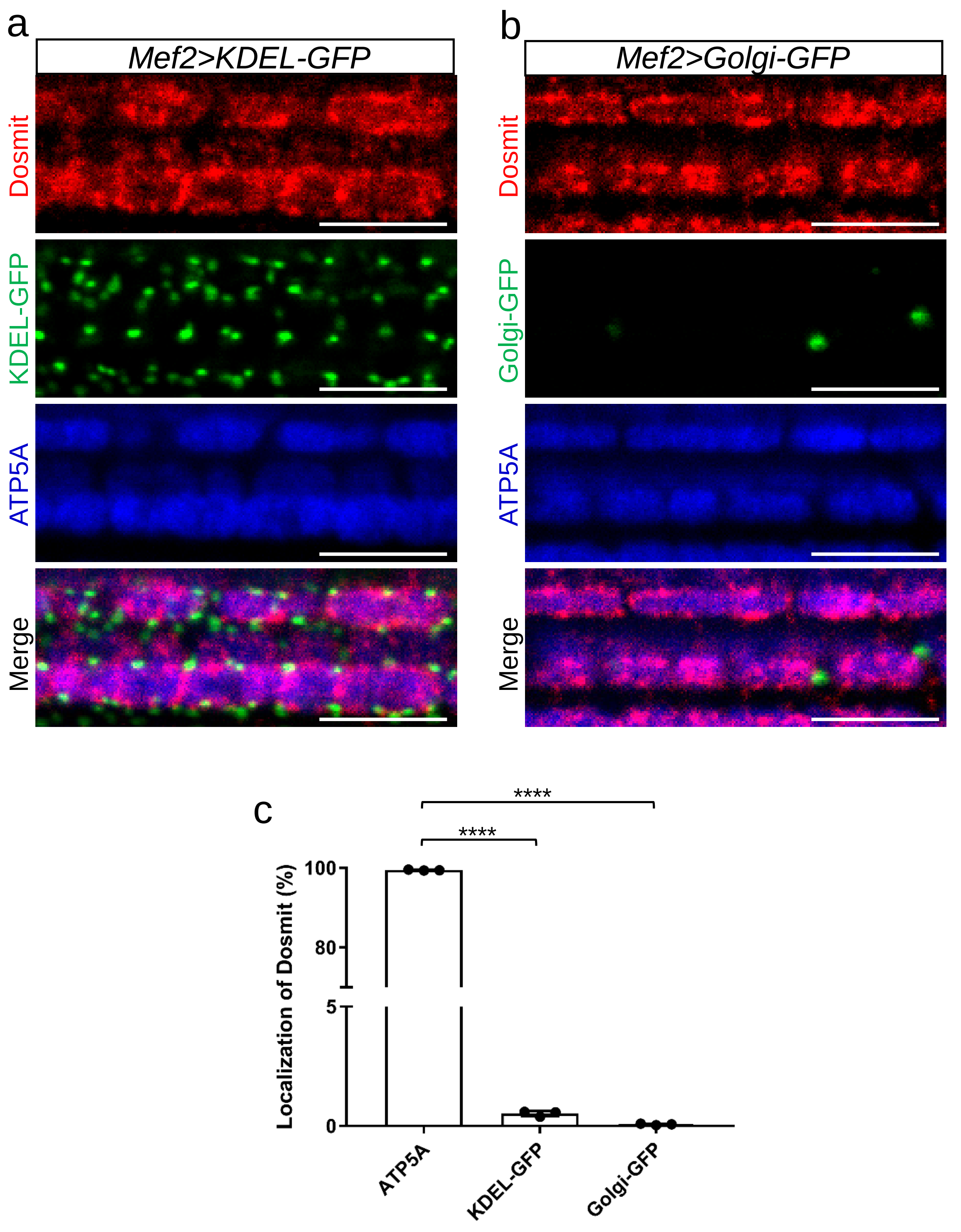

Supplementary Figure 18. ER and Golgi markers are not colocalized with Dosmit in muscle tissue.

Dosmit is mainly expressed in mitochondria and does not colocalize with **(a)** KDEL-GFP or **(b)** Golgi-GFP. Scale bar: 5 µm. **(c)** Quantification of the percentage which Dosmit colocalized with ATP5A, ER-GFP, Golgi-GFP (mean ± SD). N=3. Statistical test: Two-tailed student's t test (****$p<0.0001$). Source data are provided as a Source Data file.

Supplementary Figure 18

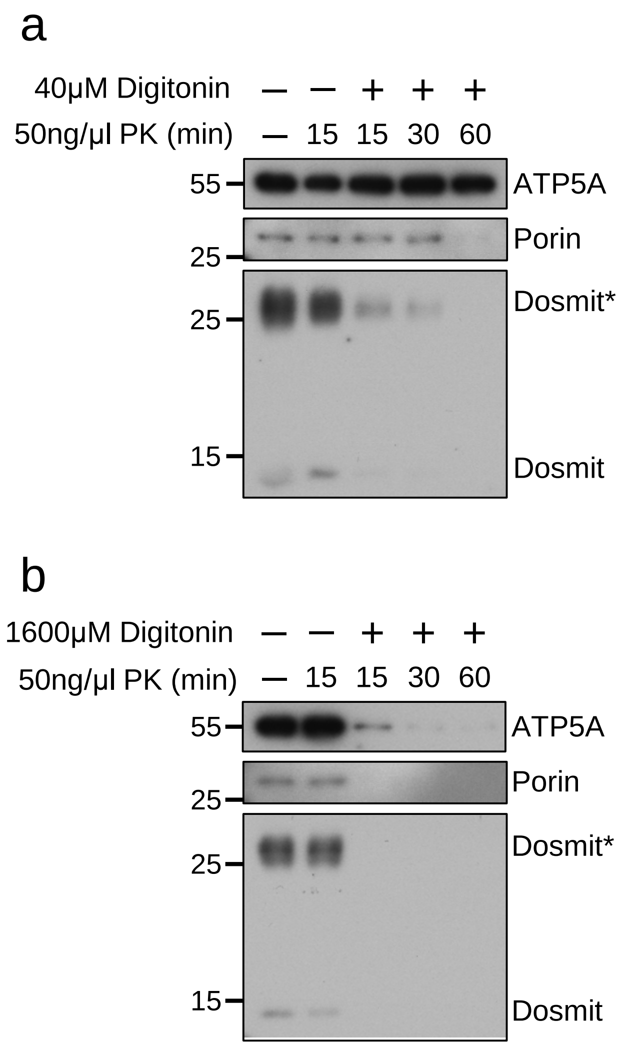

Supplementary Figure 19. Dosmit protein and an outer mitochondrial protein, Porin, are simultaneously digested with proteinase K for 60 min in digitonin-treated S2 cells. **(a)** In S2 cells treated with a low titer of digitonin, Dosmit and Porin proteins are completely digested when treated with proteinase K (PK) for 60 min. **(b)** Porin and an inner membrane protein (ATP5A) are completely digested in 15 min with a higher titer of digitonin. Source data are provided as a Source Data file.

Supplementary Figure 19

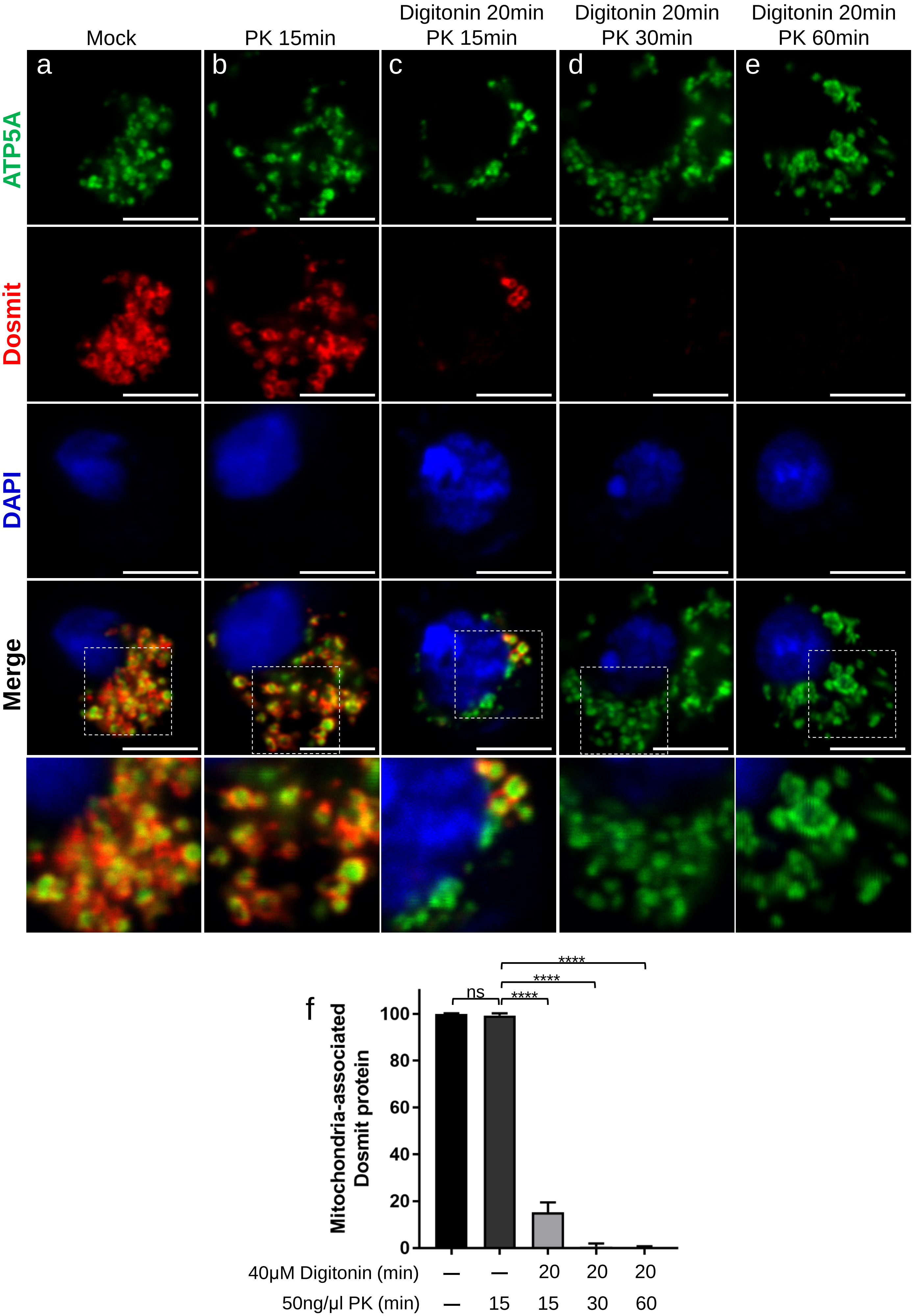

Supplementary Figure 20

Supplementary Figure 20. Dosmit protein is digested faster than ATP5A when S2 cells are treated with digitonin/proteinase K.
(a) An S2 cell without digitonin/proteinase K (PK) treatment. **(b)** An S2 cell treated with PK only for 15 min. **(c–e)** S2 cells treated with digitonin for the same length of time, but with PK for 15, 30, or 60 min. Scale bar: 5 µm. **(f)** Percentage of Dosmit-associated mitochondria in each treatment (mean ± SD). N=492, 513, 134, 221 and 398 from left to right bars. Statistical test: Two-tailed student's t-test (****$p<0.0001$; ns: $p=0.1759$). Source data are provided as a Source Data file.

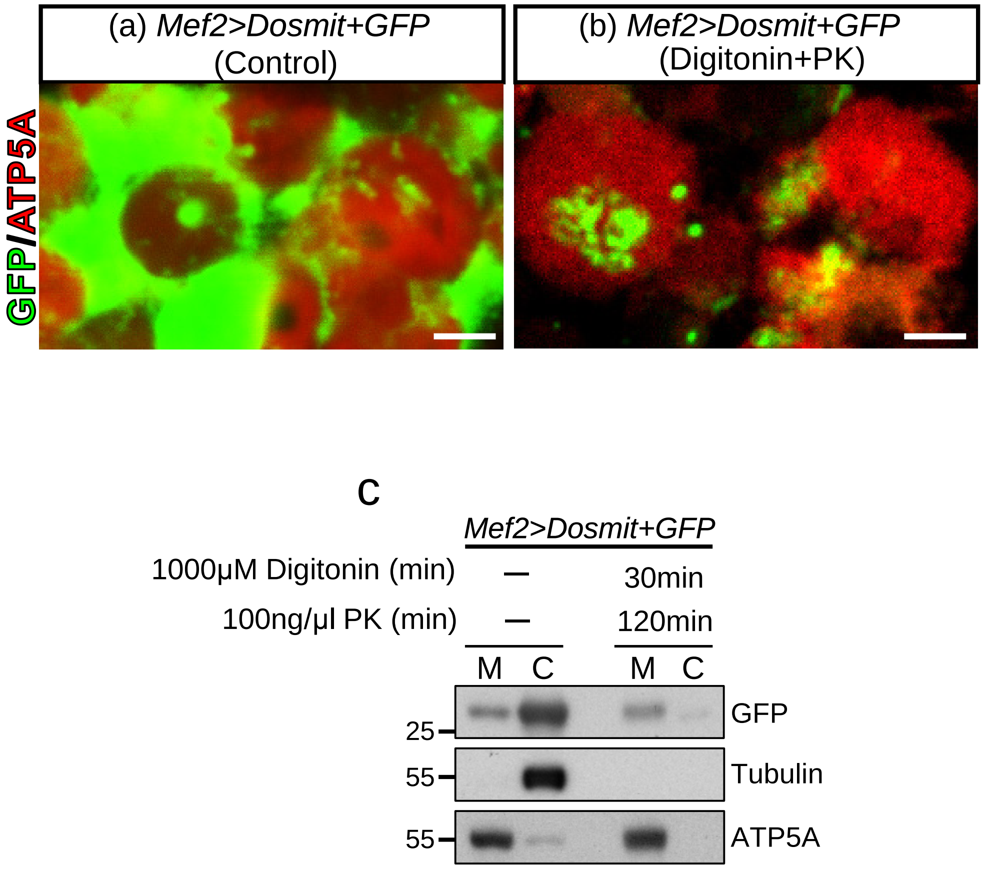

Supplementary Figure 21. Cytosolic GFP remains within mitochondria after digitonin/proteinase K treatment.

(a) *Mef2>Dosmit+GFP* muscle not treated with digitonin and proteinase K (PK). **(b)** Cytosolic GFP was digested when treated with digitonin and proteinase K. Scale bar: 2.5 μm. **(c)** Western blot showing GFP in the mitochondrial fraction (M) of flight-muscle homogenates under digitonin and proteinase K treatment from flies ectopically expressing Dosmit (*Mef2>GFP+Dosmit*), the cytosoloic fraction (C) was mostly degraded, but mitochondrial fraction remained the same level. Source data are provided as a Source Data file.

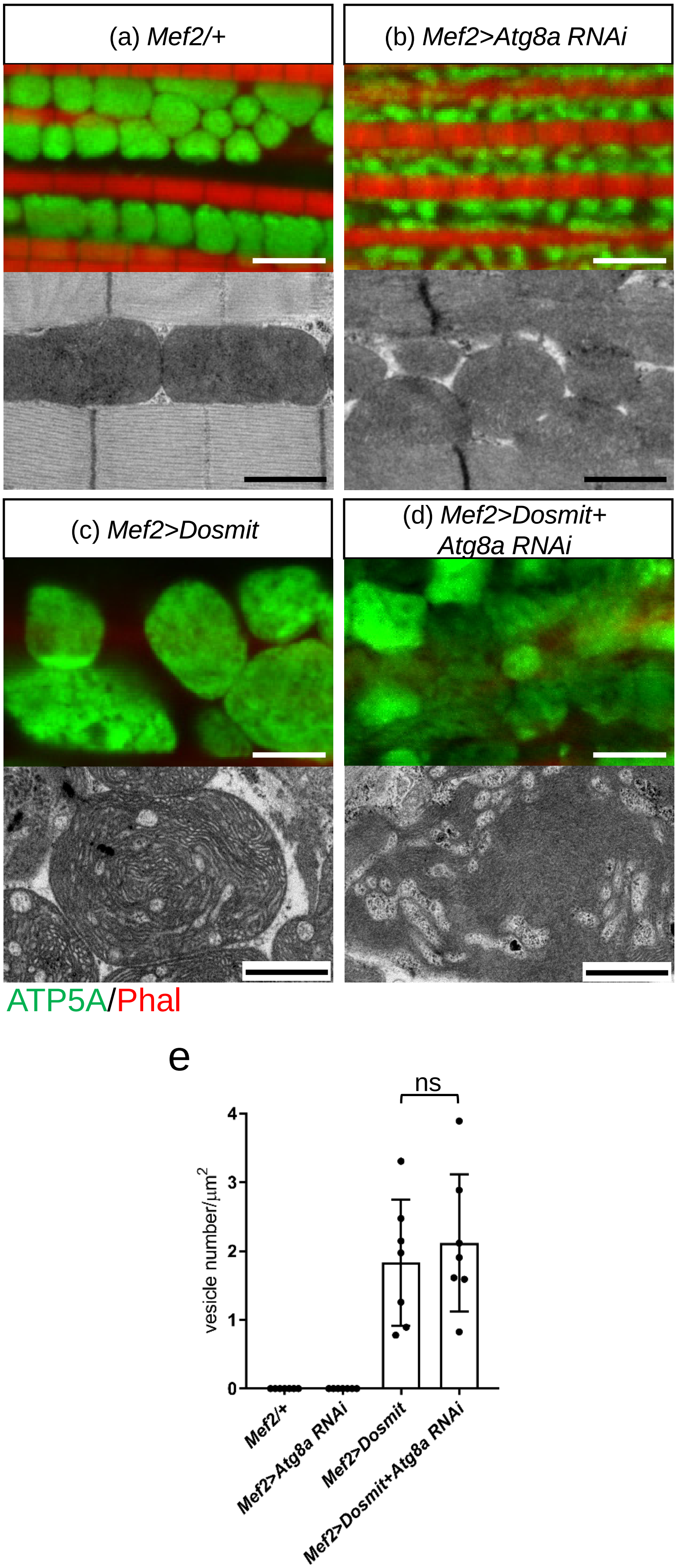

Supplementary Figure 22

Supplementary Figure 22. Atg8a knocking down could not suppress formation of intra-mitochondrial vesicles.

(a) Mitochondria morphology of *Mef2/+*, **(b)** *Mef2>Atg8a RNAi* , **(c)** *Mef2>Dosmit*, and **(d)** *Mef2>Dosmit+Atg8a RNAi*. ATP5A: green; Phalloidin: red. Scale bar: 5μm (fluorescence images), 1μm (EM images). **(e)** Quantification of intra-mitochondrial number (mean ± SD). N=7. Statistical test: Two-tailed Mann–Whitney *U* test (ns: p=0.8048). Source data are provided as a Source Data file.

Purpose	Primer name	Primer sequence
UAS-Dosmit transgenic fly	UAS_fu_Dosmit_CDS-F primer	AGGGAATTGGGAATTCATGGAGCCCATATCACATCTG
	UAS_fu_Dosmit_CDS-R primer	CCTCGAGCCGCGGCCGCCGATTTGAATTTATGTTTGGGC
UAS-Dosmit KD transgenic fly	D_Dosmit_miR-1-1	GGCAGCTTACTTAAACTTAATCACAGCCTTTAATGTGCTGCCCAATTACTTGTCACGTTAAGTTAATATACCATATC
	D_Dosmit_miR-1-2	AATAATGATGTTAGGCACTTTAGGTACGCTGCCCAATTACTTGTCAAGTTAGATATGGTATATTAACTTAACGT
	D_Dosmit_miR-2-1	GGCAGCTTACTTAAACTTAATCACAGCCTTTAATGTCAACAAGCAGACTGGAGACCACTAAGTTAATATACCATATC
	D_Dosmit_miR-2-2	AATAATGATGTTAGGCACTTTAGGTACCAACAAGCAGACTGGAGACAACTAGATATGGTATATTAACTTAGTGG
	Mir6.1_ 5' EcoRI/BglII	GGCGAATTCCGCCAGATCTTTTAAAGTCCACAACTCATCAAGGAAAATGAAAGTCAAAGTTGGCAGCTTACTTAAACTTA
	Mir6.1_ 3' NotI/BamHI	GGCCGCGGCCGCACGGATCCAAAACGGCATGGTTATTCGTGTGCCAAAAAAAAAAAAAATTAAATAATGATGTTAGGCAC
Cas9_Dosmit-2-1 transgenic fly	Cas9_Dosmit-KO_gRNA-R2	AAACAATTACTTGTCAAGTCTGCC
	pCR2_3xp3-RFP HR fu Dosmit-KO U-F	CGCCAAGCTTGGTACCCACGCTCTCCAGCAGATTGCCGTACTGCATGGCA
	pCR2_3xp3-RFP HR fu Dosmit-KO U-R	GACCAGACAAACCACACTAGTCAGCGAGGACTTCACCAGATGTGATATGG
	pCR2_3xp3-RFP HR fu Dosmit-KO D-F	GTGGTTTGTCTGGTCCTCGAGTAAGCTCTCCTGTAAGTTTGGGCAATGAG
	pCR2_3xp3-RFP HR fu Dosmit-KO D-R	AATTGGGCCCTCTAGAGCCAACCAATCCTTGACTGCAAACGAAACCAGGT
Cas9_Opa1-3xHA transgenic fly	Cas9_Opa1 stop-3xHA_F1	CTTCGGGTGTTGTGTCTTTGGTCA
	Cas9_Opa1 stop-3xHA_R1	AAACTGACCAAAGACACAACACCC
	pBluescrip fu Opa1_3xHA_U-F1	CGGGCCCCCCCTCGAGGTTGCAGCAGGAATTCATATCGCTGATGGA
	pBluescrip fu Opa1_3xHA_U-R1	GCCCGCATAGTCAGGAACATCGTATGGGTACTTTTCCTGATTAAGTGAATTGATGAACTC
	pBluescrip fu Opa1_3xHA_U-R2	TCCTGCATAGTCCGGGACGTCATAGGGATAGCCCGCATAGTCAGGAACATCGTATGGGTA
	pBluescrip fu Opa1_3xHA_U-R3	AGCGTAATCTGGAACGTCATATGGATAGGATCCTGCATAGTCCGGGACGTCATAGGGATA
	pBluescrip fu Opa1_3xHA_D-F1	GTTCCAGATTACGCTTAGTGTGCGATGACCAAAGACACAACACCC
	pBluescrip fu Opa1_3xHA_D-R1	TGGCGGCCGCTCTAGAGCAGCTAGACTAAGCGGAGGATCGGAGTGG
Cas9_Drp1-2xHA-Bac transgenic fly	Cas9_Drp1 stop-2xHA_F1	CTTCGGGGATTATTAAGTCGAAAC
	Cas9_Drp1 stop-2xHA_R1	AAACGTTTCGACTTAATAATCCCC
	pCR2_2xHA_Bac-3xp3 eGFP fu Drp1_U-F1	ATCCACTAGTGCTAGCCCGCACACCAACGTGATGGTGGAGAACATT
	pCR2_2xHA_Bac-3xp3 eGFP fu Drp1_U-R1	AACATCGTATGGGTACCACATGTGTGTCTCGCGGATTTCGCTGAT
	pCR2_2xHA_Bac-3xp3 eGFP fu Drp1_D-F1	TCTTTCTAGGGTTAAGCTGGTCAGCGATTCGAGCAACTCGTTTCG
	pCR2_2xHA_Bac-3xp3 eGFP fu Drp1_D-R1	TAGATGCATGCTCGAGCTGCTGCAGTGGCATGAGAAGGCCTTCGCC
Cas9_Marf-2xHA-Bac transgenic fly	Cas9_Marf stop-2xHA_F1	CTTCCCGCAGTAGTGGAACAGTAG
	Cas9_Marf stop-2xHA_R1	AAACCTACTGTTCCACTACTGCGG
	pCR2_2xHA_Bac-3xp3 eGFP fu Marf_U-F1	ATCCACTAGTGCTAGCGAATTGCCAGAACCTCTGCGCCGACTTCCA
	pCR2_2xHA_Bac-3xp3 eGFP fu Marf_U-R1	AACATCGTATGGGTACTGCGGCGATATATAGTTGTGCTCGAAGAT
	pCR2_2xHA_Bac-3xp3 eGFP fu Marf_D-F1	TCTTTCTAGGGTTAATCGAACAGTAGTCGGTTCGCTGACCGTGGG
	pCR2_2xHA_Bac-3xp3 eGFP fu Marf_D-R1	TAGATGCATGCTCGAGTATTTACTGATAACGGATGCTAATGGAAGC
Rab32-D type-3xHA (for cell transfection)	pAc5.1-3xHA Rab32-D type-CDS_EcoRI-F	TGGTGGAATTCATGACACTAAGATCCAAAGCCATCA
	pAc5.1-3xHA Rab32-D type-CDS_XhoI-R	GGGTACTCGAGGCAGGAACACTTGTTCTTGGCATCC
Hsc70-5-3xHA (for cell transfection)	pAc5.1-3xHA fu Hsc70-5-CDS_EcoRI-F	CAGTGTGGTGGAATTCATGCTGCGCGTACCCAAGTTTCTGCCCCGT
	pAc5.1-3xHA fu Hsc70-5-CDS_XhoI-R	CGTATGGGTACTCGAGGTTCTTCTCTTCCTTCTTGGCTTCGCCGGA
Dosmit-wt-3xHA (for cell transfection)	pAc5.1-3xHA fu Dosmit-wt_EcoRI-F	CAGTGTGGTGGAATTCATGGAGCCCATATCACATCTGGTGAAGTC
	pAc5.1-3xHA fu Dosmit-wt_XhoI-R	CGTATGGGTACTCGAGCTTCTTGATGACAATTGGTCCGACGTTGT
Dosmit-△CDGSH-3xHA transgenic fly	pAc5.1-3xHA fu Dosmit-wt_EcoRI-F	CAGTGTGGTGGAATTCATGGAGCCCATATCACATCTGGTGAAGTC
	pAc5.1-3xHA fu Dosmit-△CDGSH_XhoI-R	CGTATGGGTACTCGAGGTAGGGCCAGTTCTTGGTCTTCCAG
Dosmit-△N-3xHA transgenic fly	pAc5.1-3xHA fu Dosmit-△N_EcoRI-F	CAGTGTGGTGGAATTCATGGATTGGTTGGCCCTGATCCCACCCAC
	pAc5.1-3xHA fu Dosmit-wt_XhoI-R	CGTATGGGTACTCGAGCTTCTTGATGACAATTGGTCCGACGTTGT
mCisd1-3xHA (for transgenic fly and cell transfection)	pAc5.1-3xHA fu mCisd1-CDS_EcoRI-F	CAGTGTGGTGGAATTCATGGGCCTCAGCTCCAACTCCGCT
	pAc5.1-3xHA fu mCisd1-CDS_XhoI-R	CGTATGGGTACTCGAGGGTTTCCTTTTTCTTGATGATCAGAGGTC
mCisd2-3xHA (for transgenic fly and cell transfection)	pAc5.1-3xHA fu mCisd2-CDS_EcoRI-F	CAGTGTGGTGGAATTCATGGTCCTGGACAGCGTGGCCCGCATCGT
	pAc5.1-3xHA fu mCisd2-CDS_XhoI-R	CGTATGGGTACTCGAGTACTTCTTTCTTCTTCAGGATGAGAGGAC
N-mCisd2-mCisd1-3xHA (for transgenic fly and cell transfection)	pAc5.1-3xHA fu N-mCisd2-mCisd1_R	GGCCGCGATCCACTCTGAAACTGTGAGGCGGGCGAACCCGGTGAT
	pAc5.1-3xHA fu N-mCisd2-mCisd1_F	GAGTGGATCGCGGCCGTCACCTTTGCTGCTGGCACAGCCGCTCTC

Supplementary Table 1. Primer information

Supplementary Table 1
